# A Light-Activated Antimicrobial Surface Is Active Against Bacterial, Viral and Fungal Organisms

**DOI:** 10.1038/s41598-017-15565-5

**Published:** 2017-11-10

**Authors:** Tim Walker, Melisa Canales, Sacha Noimark, Kristopher Page, Ivan Parkin, Jane Faull, Manni Bhatti, Lena Ciric

**Affiliations:** 1 Healthy Infrastructure Research Group, Department of Civil, Environmental & Geomatic Engineering, University College London, London, UK; 2 Materials Chemistry Centre, Department of Chemistry, University College London, London, UK; 3 Department of Biological Sciences, Birkbeck College, London, UK

## Abstract

Evidence has shown that environmental surfaces play an important role in the transmission of nosocomial pathogens. Deploying antimicrobial surfaces in hospital wards could reduce the role environmental surfaces play as reservoirs for pathogens. Herein we show a significant reduction in viable counts of *Staphylococcus epidermidis*, *Saccharomyces cerevisiae*, and MS2 Bacteriophage after light treatment of a medical grade silicone incorporating crystal violet, methylene blue and 2 nm gold nanoparticles. Furthermore, a migration assay demonstrated that in the presence of light, growth of the fungus-like organism *Pythium ultimum* and the filamentous fungus *Botrytis cinerea* was inhibited. Atomic Force Microscopy showed significant alterations to the surface of *S*. *epidermidis*, and electron microscopy showed cellular aggregates connected by discrete surface linkages. We have therefore demonstrated that the embedded surface has a broad antimicrobial activity under white light and that the surface treatment causes bacterial envelope damage and cell aggregation.

## Introduction

Infections originating in hospitals (nosocomial infections, or healthcare associated infections) are a major problem in modern healthcare. A 2011 survey found the prevalence of nosocomial infections in Scotland to be 4.6%^1^. Up until the last decade, it was believed that contamination of environmental surfaces played a minimal role in transmission of nosocomial infections and efforts to control transmission focused on effective hand hygiene of healthcare workers^2^. However, there is now increasing evidence that strongly suggests that environmental surfaces are a major factor in transmission of nosocomial pathogens such as Norovirus, *Clostridium difficile*, *Acinetobacter* spp., methicillin-resistant *Staphylococcus aureus* (MRSA), vancomycin-resistant *enterococci* (VRE), and *Pseudomonas aeruginosa* ^3,4^.

Work has previously been carried out^5,6^ to incorporate a combination of two photosensitizers and nanogold into a soft polymer to be employed in hospital touch surfaces (e.g mobile phone covers, computer keyboards and hand dryers) to reduce nosocomial infection transmission. Similar studies have been carried out to translate this antimicrobial technology for catheter applications to reduce catheter-associated infections, using low power red laser light to activate the antimicrobial activity^7,8^.

Photosensitizers are molecules that utilise light energy to elicit a toxic affect upon living systems. The photosensitizer molecule is excited, with light of a wavelength at its absorbance peak, to a singlet state. The energy is either released as fluorescence or the molecule then crosses to a lower energy triplet state where it can undergo one of two reaction pathways. The Type I pathway involves electron transfer between the triplet state photosensitizer molecule and nearby molecules, such as water or biological molecules, to produce a wide range of reactive oxygen species (ROS) and radical ions. The Type II process involves energy transfer between the triplet state photosensitizer and molecular oxygen, which has a triplet ground state, resulting in the generation highly reactive singlet oxygen. The radical products and singlet oxygen are highly reactive and are able to attack the cellular membrane, essential enzymes, and cellular nucleic acids, leading to cell death by oxidative stress^9^.

Herein, we present the assessment of the antimicrobial activity of the light-activated methylene blue-crystal violet-nanogold silicone surface tested against unicellular microorganisms: *Staphylococcus epidermidis*, *Saccharomyces cerevisiae*, and MS2 Bacteriophage as model organisms for bacteria, yeasts, and viruses, respectively. The antimicrobial activity of the surface was also tested against filamentous organisms: *Botrytis cinerea* and *Pythium ultimum*. Finally, microscopy and spectrometry were used to determine whether the generation of exogenous ROS by the photosensitizer-based surface caused damage to the bacterial cell envelope, as proposed by Dahl, *et al*.^10^.

## Results

### Materials development

**Figure 1:**
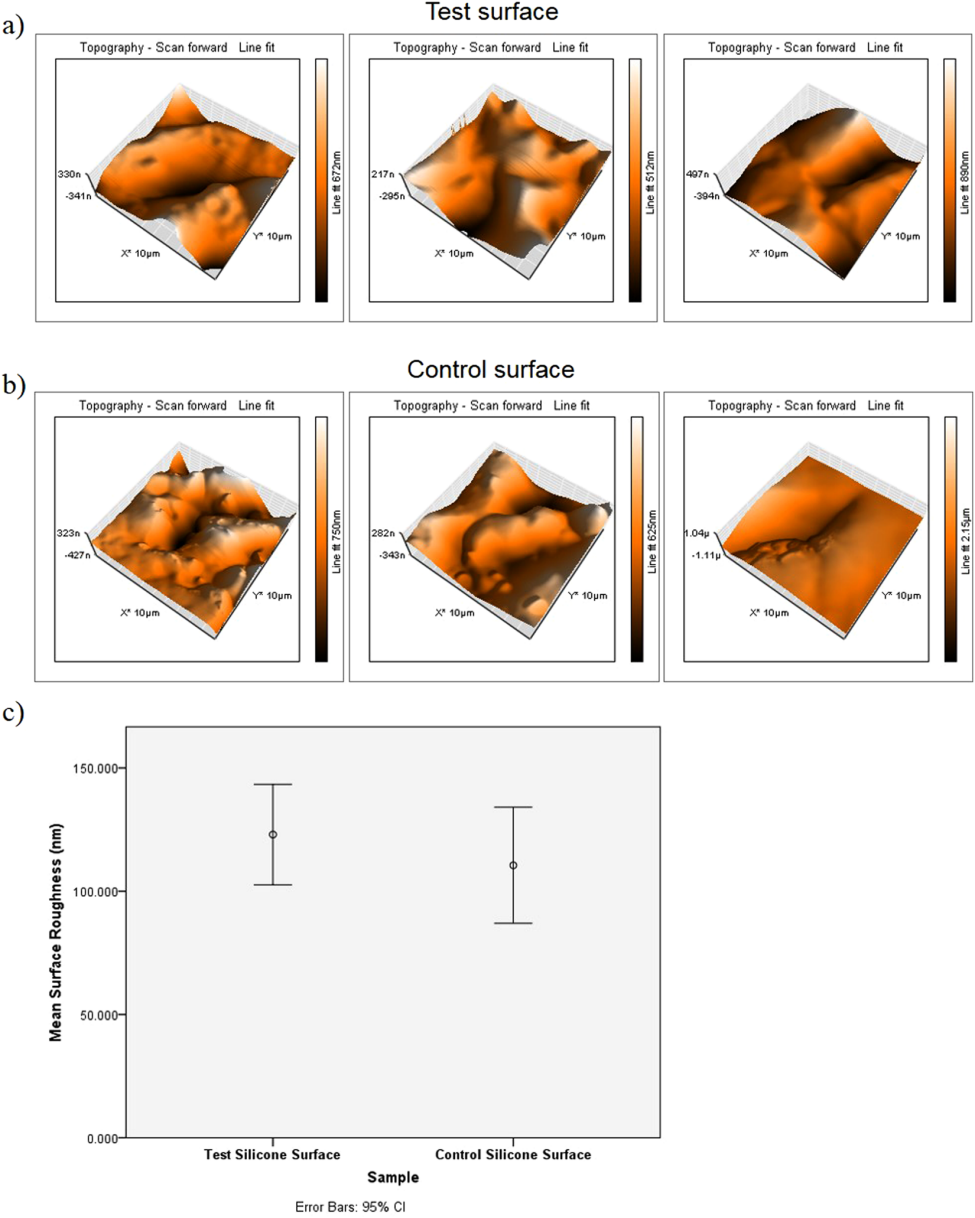
Atomic force microscope topographical scans of (**a**) three representative 10 μm^2^ sections of the test surface and (**b**) three representative 10 μm^2^ sections of the control surface. (**c**) Shows the error plot graph comparing the mean surface roughness taken from 10 μm^2^ AFM topographical scans of the test and the control surfaces (n = 30).

UV-Vis spectroscopy indicated strong absorption bands characteristic of the two dyes, with peak maxima at 590 nm typical of crystal violet and at 650 nm typical of methylene blue. Table 1 shows the mean absorbance values for the two dyes after the surfaces were incubated in PBS for 90 minutes in light and darkness which confirmed that a small amount of the dyes does leach out from the test surfaces. The results of comparisons of the mean area roughness of the test surface with the control surface is show in Fig. 1. It is demonstrated that the incorporation of the methylene blue, crystal violet, and the gold nanoparticles did not alter the topography of the silicone.

**Table 1:** Assay of dye leaching from test surface in PBS. Mean and standard deviation for absorbance at 590 nm and 651 nm are shown (n = 3).

Sample	Absorbance of PBS at 590 nm	Absorbance of PBS at 651 nm
PBS containing test surface kept in light	0.003 (0.002)	0.003 (0.004)
PBS containing test surface kept in darkness	0.013 (0.006)	0.010 (0.004)

### Quantitative assessment of antimicrobial activity

**Figure 2:**
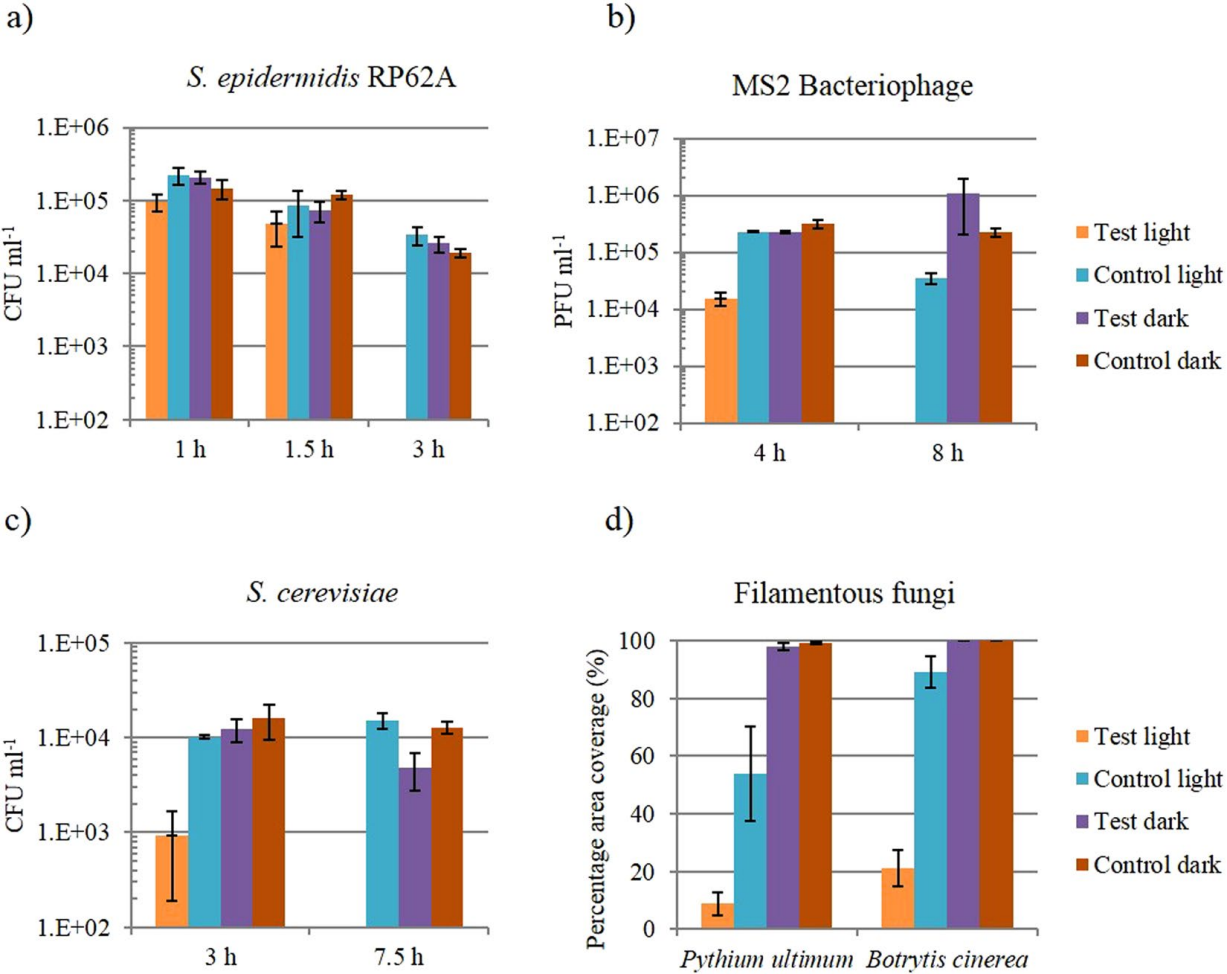
Activity of antimicrobial surfaces against (**a**) *S*. *epidermidis* (n = 3), (**b**) MS Bacteriophage (n = 3), (**c**) *S. cerevisiae* (n = 3) and (**d**) filamentous fungi (n = 4). Test light refers to the surfaces containing methylene blue-crystal violet-nanogold and control dark refers to a control surface composed of silicone only in the absence of light.

After 3 hours light exposure there was a ≥2.92 log_10_ reduction of *S*. *epidermidis* to below the detection limit of 400 CFU/ml (Fig. 2a). This result was statistically significant (P = 0.03) against the starting inoculum and all other samples. At prior time points of 1 hour and 1.5 hours, there was no significant reduction in the *S*. *epidermidis* population. From a starting inoculum of 3 × 10^6^ PFU/ml the test light MS2 Bacteriophage was reduced by 2.33 log_10_ after 4 hours and ≥4.61 log_10_ after 8 hours below the below the detection limit of 100 PFU/ml (Fig. 2b). Figure 2c shows that, after 3 hours there was a 1.5 log_10_ (P = 0.05) reduction in the *S*. *cerevisiae* population and after 7.5 hours, the reduction was below the detection limit of 400 CFU/ml. This reduction is significant against the starting inoculum and the other sample populations (P = 0.03). Figure 2d shows the effect of the antimicrobial surfaces on the filamentous organisms. The mean coverage of the test light surface by *P*. *ultimum* was 8.75% compared to a coverage of 99.25% on the control square in the dark, a statistically significant effect (P < 0.001). The mean coverage of the test light surface by *B*. *cinerea* was 21% and coverage of the control surfaces in the dark was 100%, again, a statistically significant effect (P < 0.001).

### Qualitative assessment of antimicrobial activity

**Figure 3:**
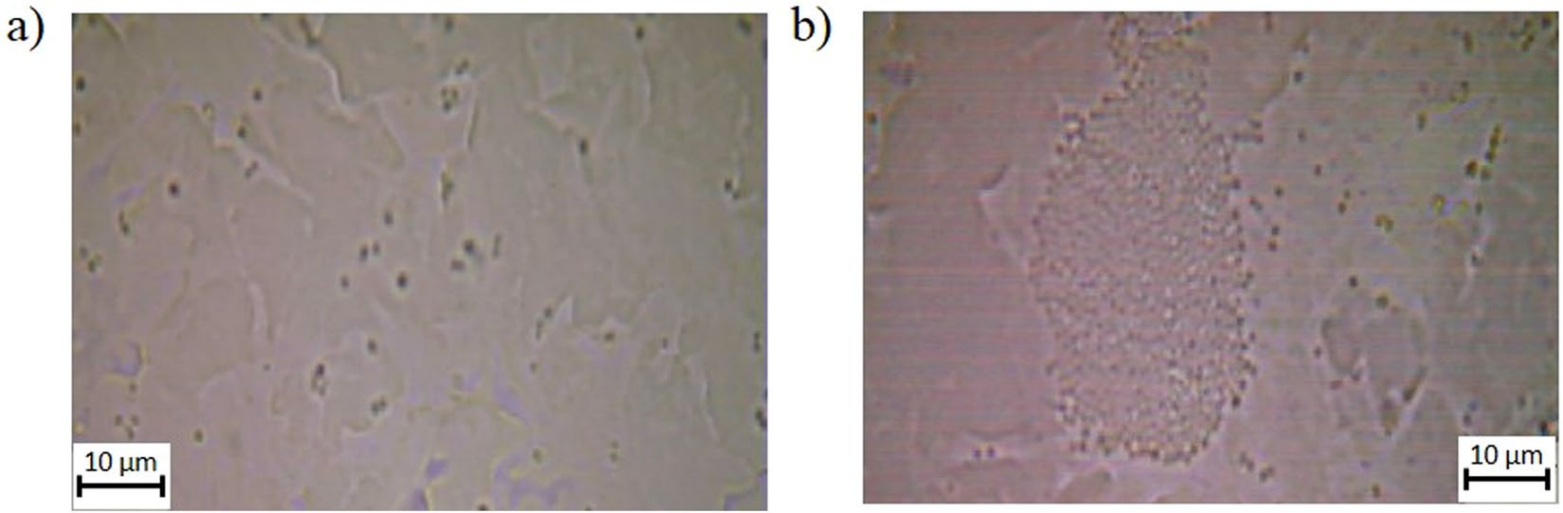
Light microscope images of *S*. *epidermidis* on surfaces. (**a**) A 100x optical microscope representative image of *S*. *epidermidis* aqueous suspension on a control surface and (**b**) shows *S*. *epidermidis* on a test surface.

Observation of *S*. *epidermidis* using optical microscopy showed that interaction between the cells and the surface occurs primarily in the indentations on the surface (Fig. 3). However, it is clear that on the control surface (Fig. 3a) the cells are evenly spread in small groups but on the antimicrobial test surface (Fig. 3b) the cells are in large aggregations.

**Figure 4:**
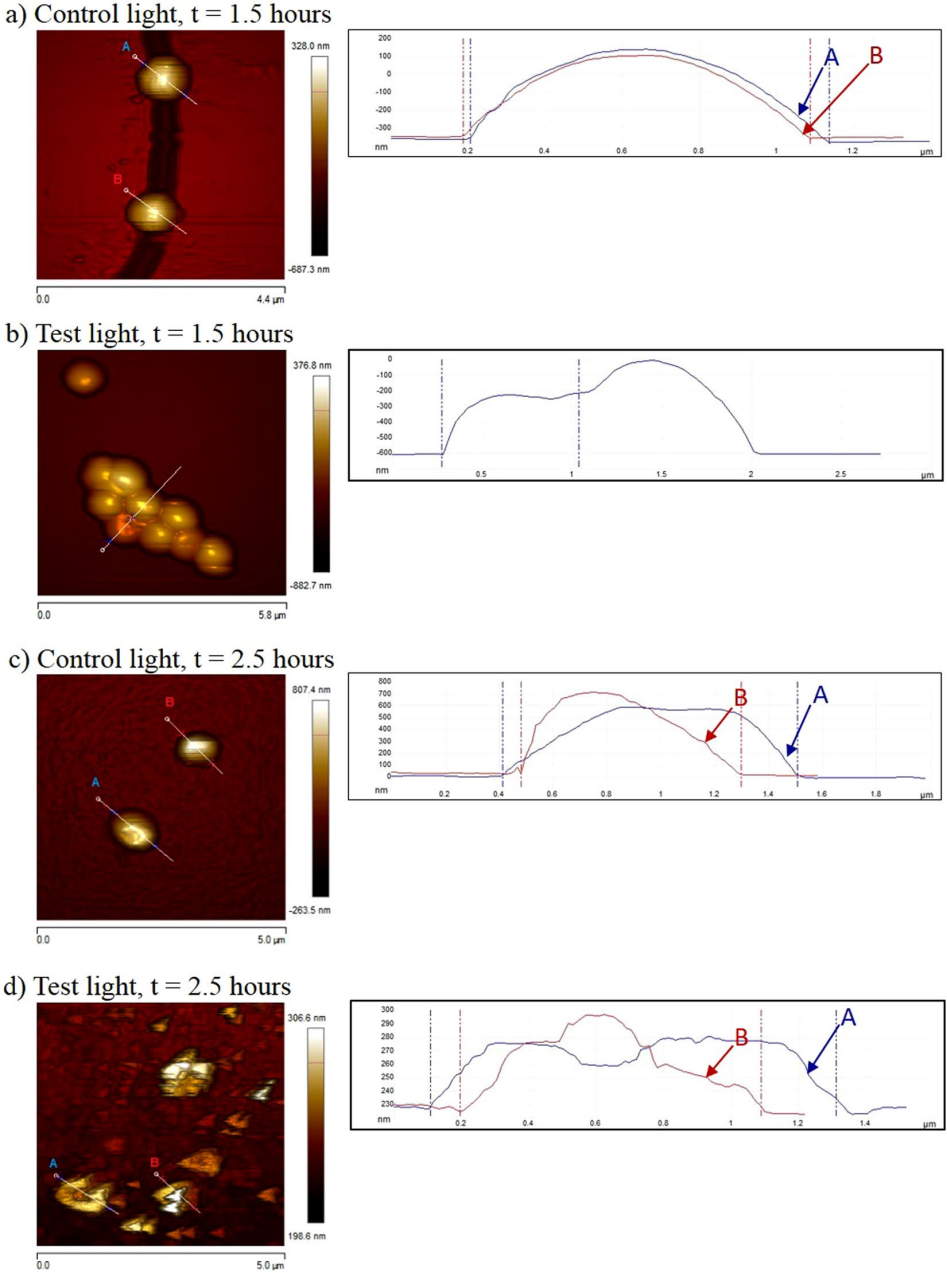
Height sensor AFM images and their cross-sectional analyses of *S*. *epidermidis* cells on test and control surfaces in the presence of light. (**a**) Shows the cells after 1.5 hours of treatment on the control surface (scale bar = 4.4 μm); (**b**) shows the cells after 1.5 hours of treatment on the test surface (scale bar = 5.8 μm); (**c**) shows the cells after 2.5 hours of treatment on the control surface (scale bar = 5.0 μm); and (**d**) shows the cells after 2.5 hours of treatment on the test surface (scale bar = 5.0 μm).

Figure 4 shows height sensor images of *S*. *epidermidis* after various lengths of treatment on test and control surfaces in the presence of light. On the control surfaces at 1.5 hours, the cell surfaces are generally smooth with few irregularities (Fig. 4a). Most of the cells that were treated on the test surface in the light for 1.5 hours appeared as smooth spheroids (cells A & B in Fig. 4a), which is typical coccal morphology. Some cells from the test surface sample were seen to have discrete indentations on their surfaces, which cross section analysis showed to be 10 nm and 20 nm deep, respectively (Fig. 4b). These indentations suggest that the surface of the cell has lost structural integrity due to damage. Control surface cells were smooth spheroids; with cross section analysis also showing the surfaces to be smooth, suggesting that the cell envelope is intact.

*S*. *epidermidis* was subsequently imaged on the AFM after 2.5 hours treatment on the surfaces in the presence of light. As expected, the cells treated on the control surface appear as typical spheroids (cells A & B in Fig. 4c). In comparison, the cells that have been treated for 2.5 hours on the test surface all appeared very irregularly shaped (Fig. 4d). The “horse-shoe” appearance of cells A and B in Figure 4d suggests that the cell has lysed. Cross section analysis revealed that the surface of the cells is very rough, further suggesting that the cell envelope has sustained damage. The more extensive surface damage is seen in the samples treated in the presence of light on the test surface for 2.5 hours (Fig. 4d) compared to 1.5 hours (Fig. 4b) is in accordance with the exponential kill effect observed through the CFU counts of *S*. *epidermidis* after test surface treatment (Fig. 2a).

**Figure 5:**
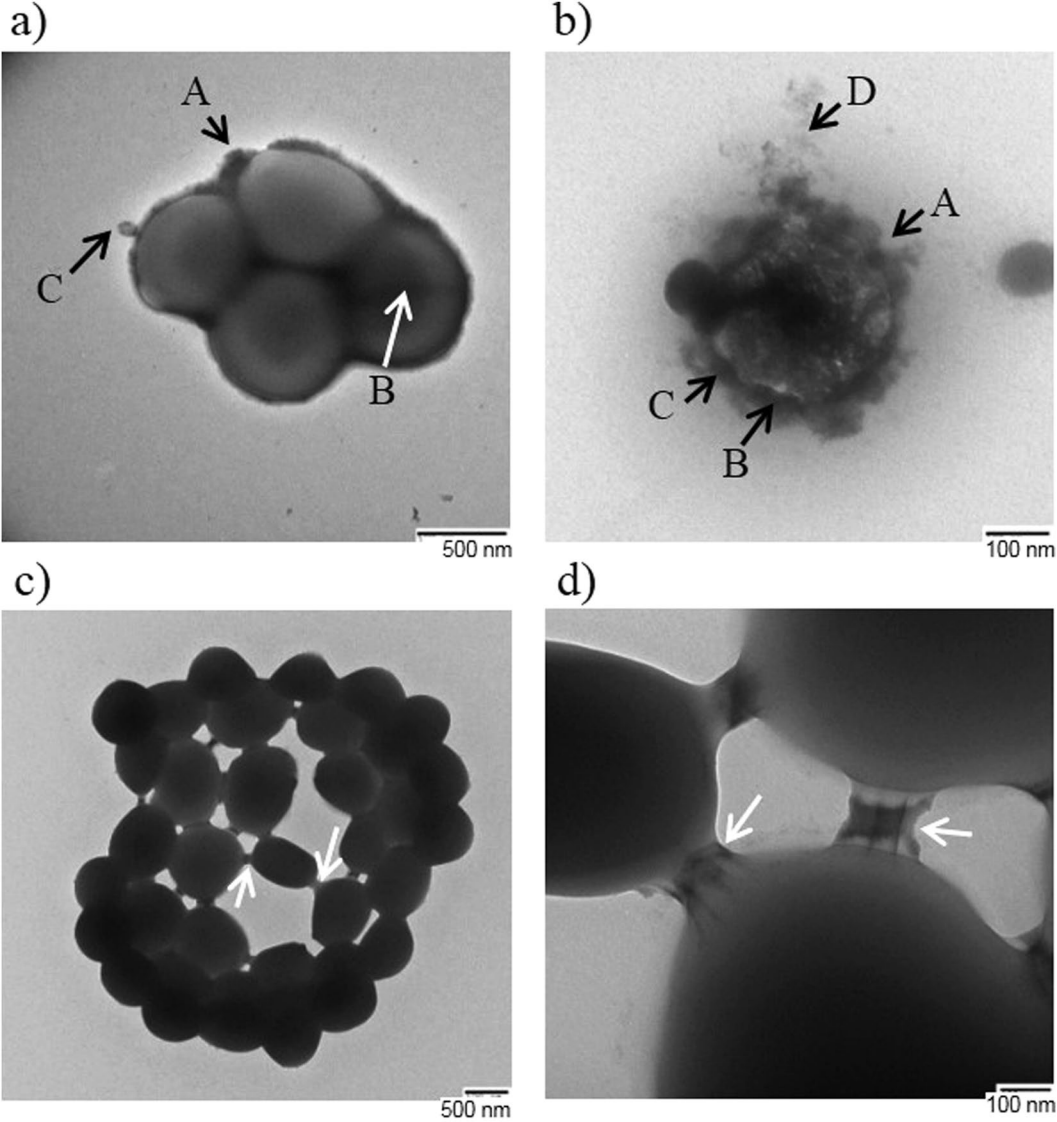
Transmission Electron Microscopy (TEM) of *S*. *epidermidis* after surface treatment. (**a**) Shows *S*. *epidermidis* cells treated with 2.5 hrs light on control surface (scale bar = 500 nm), (**b**) shows cells after 2.5 hrs treatment on test surface (scale bar = 100 nm), (**c**) shows a cluster of treated cells (2.5 hrs light treatment on test surface) showing links between cells (scale bar = 500 nm); and (**d**) shows a closer TEM image of the links between cells from (**c**) (scale bar = 100 nm).

Transmission electron microscopy (TEM) was used to further analyse the cells. TEM images of the *S. epidermidis* samples were taken in an attempt to observe the nature of the interactions between aggregated cells. Figure 5a shows *S*. *epidermidis* after 2.5 hours treatment on the control surface in the presence of light in a typical “grape like” clustering. The majority of cells that had been treated on the control surface had a healthy appearance, with typical coccal morphology, and were in small groupings of no more than five cells. Some cells were surrounded by a thick electron-lucent capsule layer (Fig. 5a, arrow B), which was distinguishable from the thinner electron-dense peptidoglycan cell wall (Fig. 5a, arrow A). Some cells had no obvious capsule layer and peptidoglycan cell wall appeared rough (Fig. 5a, arrow A). Some of the cells observed had vesicles on the surface or septa (Fig. 5a, arrows B & C). Figure 5 b,c, d show *S*. *epidermidis* after 2.5 hours treatment on the test silicone surface in the presence of light. As shown by the quantitative data, not all cells are lysed until 3 hours of treatment. Figure 5b shows a single *S*. *epidermidis* cell that has lysed. Arrows A, B & C highlight indentations in the cell membrane. Arrow D highlights an electron-dense, irregular shaped substance, which is surrounding the cell. As seen in the other microscopy images taken, a large number of the cells treated on the test surface were in aggregations. Figure 5c shows that cells treated on the test surface with light for 2.5 hours have aggregated and the cells appear to be connected by discrete bridges that are linking opposing cells at their cell walls. Figure 5c shows a large congregation of *S*. *epidermidis* cells that are in a spherical arrangement. The arrows highlight sites of linkage between opposing cells in the structure. The linkages appear as bridges connecting the cell wall of each cell and Fig. 5d is a close up image of some of the linkage sites seen in Fig. 5c. The arrow highlights two rod-like structures that connect both cell envelopes. The rods are approximately 125 nm long, 5 nm wide, and are 45 nm apart.

## Discussion

Crystal violet-coated, methylene blue and 2 nm gold nanoparticle encapsulated medical grade silicone polymers were developed using a two-step coating strategy as described previously^5,7^. The materials were first immersed in a methylene blue saturated gold nanoparticle: acetone swelling solution for 72 h, to facilitate optimal incorporation of the gold nanoparticles^11^. Subsequently, after air-drying and washing the polymers were exposed to an aqueous crystal violet solution such that strong uptake of the dye was achieved on the polymer surfaces, for high surface generation of ROS in the environs of contaminating microorganisms^7^.

*S*. *epidermidis* was used in this study as it is a very common coloniser of catheter materials, which can cause systemic nosocomial infections^12^. The data presented show that a significant kill of *S*. *epidermidis* cells occurs only after 1.5 hours. This would suggest that the antimicrobial activity of the test surface follows an exponential regression, possibly due to damage of the cell envelope due to production of exogenous ROS by the test surface has to accumulate over time before it leads to a loss in cell viability. During the initial stages of exposure, most of the exogenous ROS are likely quenched through interaction with the peptidoglycan cell wall protecting the cytoplasmic membrane. However, over time, there is an accumulation of radical products from the interaction of ROS with the cell wall, which can attack the cytoplasmic membrane. Furthermore, sustained ROS damage to the cell wall increases its porosity^10^, allowing more exogenous ROS to reach the cytoplasmic membrane, probably leading to peroxidation of the cytoplasmic membrane, which causes cell leakage and death.

The *S*. *cerevisiae* cell wall shares many similarities with *Candida albicans* ^13^, a common yeast coloniser of catheters that can cause nosocomial infection^14^. A significant decrease in the number of viable *S*. *cerevisiae* cells takes place after 3 hours, suggesting that the yeast is less susceptible to the photosensitizer than *S*. *epidermidis*, an observation also made by Decraene *et al*.^15^ with a toluidine blue and rose bengal containing cellulose acetate surface. This may be explained by the greater thickness and complexity of the yeast cell wall providing a greater level of protection from the exogenous ROS. The simpler cross-linking in the *S*. *epidermidis* cell wall means the peptidoglycan forms a more open, crystal lattice structure, making it more porous. However, the ROS are likely quenched by the denser network of polymers in the yeast cell wall, protecting the cytoplasmic membrane. Over time, the build-up of radical products from the reaction of ROS with the cell wall, and its increased porosity allow more exogenous ROS through, will result in oxidative damage of the yeast cytoplasmic membrane and cell death.

It was also found that there was a significant reduction in *S*. *cerevisiae* after exposure to test surface in darkness after 7.5 hours. As stated in a recent review by Maley & Arbiser^16^, crystal violet dye has a documented antimicrobial and antifungal activity, which does not require light activation, and this property has been exploited clinically since the nineteenth century before falling out of favour with the discovery of antibiotics. Both the methylene blue and crystal violet dyes leach out of the silicone surface at low levels after 90 minutes submersion in PBS. It is probable therefore, that these free flowing crystal violet molecules, although at a low concentration, interact with the test organism and initiate an antimicrobial effect in the dark. It has been shown that the amount of crystal violet that leaches out of similar polyurethane polymers is ~1 μM after 24 hours^17^ and therefore, it is very unlikely that the exogenous dyes would contribute to such significant reductions in cell number in the presence of light. For example, Kondo *et al*., (2012)^18^ showed that after a 24 hour incubation period, 5 μM crystal violet is required to reduce the viable count of *S*. *cerevisiae* by 90%. The results for both the *S*. *epidermidis and S*. *cerevisiae* show that over time, the amount of cell death in the dark test samples increases slightly as the incubation time increases. Therefore, in the presence of light, there may be some cell death attributed to these free molecules. However, it has been well documented that for Gram-positive bacteria at least ~25 μM of methylene blue is needed in the presence of light for a 7 log_10_ reduction and for dark toxicity, at least 44 μM are required for ~5 log_10_ reduction^19,20^. Furthermore, it has been shown that crystal violet in the presence of light is less potent towards Gram-positive (4 log_10_) compared to Gram-negative (7 log_10_) bacteria at concentrations of 10 μM^21^, showing that again, any free crystal violet molecules are unlikely to cause any greatly significant reductions in viable cells. Regarding the gold nanoparticles, a previous study^22^ has demonstrated that 2 nm gold nanoparticles have no antimicrobial activity against bacteria when immobilised in a soft polymer surface, but rather enhance the phototoxic effect of the immobilised photosensitizers. Time-resolved electron paramagnetic resonance studies have indicated that the presence of 2 nm gold nanoparticles result in an increase of approximately 38% in dye triplet state production^8^. It can be speculated that increased triplet state yields may lead to an enhancement in the subsequent ROS generation.

Bacteriophage MS2 has a similar structure and size to important human enteric viruses, such as Norovirus, which has led to it being used extensively in research as a surrogate for these viruses when testing different disinfection techniques^23^. The present study has shown that the phage titre was significantly reduced when exposed to the test surface and white light due to inactivation the test organism though the generation of exogenous ROS. The titre of the suspension on the control surface in the presence of light was also significantly reduced compared to the other samples, as observed in a previous study of MS2 exposed to UV light because of UV leakage from lamp^24^.

Filamentous fungi are becoming increasingly common opportunistic pathogens of immunocompromised hospital patients^25^. Diverse species such as *Aspergillus* spp^26,27^., *Fusarium* spp^28^., *Mucor* spp^29^., and *Absidia* spp^30^. have been documented as causative agents of catheter-associated system infections. The results of our study show that growth of the fungus-like organism *P*. *ultimum* and the fungus *R*. *solani* (Fig. 1c) was inhibited on the test surface in the presence of light. It is possible that *P*. *ultimum* was more susceptible to the test surface because exogenous ROS were able to readily diffuse through the porous cell wall to attack the hyphal membrane. The lower porosity of the cell walls of *B*. *cinerea* may protect the hyphal membranes by quenching exogenous ROS. Differences in the melanin content of the fungal cell walls and in the fungal cell wall constituents is likely the reason for the differences observed^13^.

The images of the AFM scans confirmed that incorporation of the dyes and nanogold into the silicone does not significantly change its topography contrary to the findings of Perni *et al*.^31^ who found this to be the case only when they employed a similar swell-encapsulation-shrink method to immobilize toluidine blue into soft polymers. Optical microscopy of the control and test surfaces with *S*. *epidermidis* showed that interaction between the cells and the surfaces occurs primarily in the indentations on the surface, a possible reason for the success of *S*. *epidermidis* in colonising catheter materials.

In line with optical microscopy results, AFM images of fixed *S*. *epidermidis* that had been treated on the test surface showed that cells treated for 1.5 hours in the presence of light aggregated into large masses. Cells that had been treated for 2.5 hours on the test surface all appeared very irregularly shaped with a “horse-shoe” appearance suggesting lysis. The more extensive cell surface damage seen after 2.5 hours compared to 1.5 hours is in accordance with the earlier observation of an exponential kill effect observed through the CFU counts of *S*. *epidermidis* after test surface treatment.

TEM images of the *S*. *epidermidis* samples were taken in an attempt to observe the nature of the interactions between aggregated cells. A proposed theory to explain the appearance of discrete linkages between the cells is that they are envelope stress induced conjugative pili. *Staphylococcus* pili are not well understood, but the long and thin appearance of the rods is similar to the reported dimensions of pili of other Gram-positive species^32^. Therefore, damage to the cell envelope experienced by cells exposed to the test surface may be promoting conjugation, leading to the formation of these aggregations of cells. Another theory to explain the observation of these cell aggregations is that treatment on the test surface is promoting the formation of a biofilm. *S*. *epidermidis* produces a biofilm by secreting a molecule called polysaccharide intercellular adhesin. It is encoded by the *ica* gene cluster and causes cells to attach to each other, forming a bacterial aggregation^33^. TEM observations have further supported the theory that the test surface causes damage to the cell membrane, though it is not possible to assess the relative contributions to cell envelope damage made by the leached photosensitizers, and possibly nanogold, and by exogenous ROS, respectively, from the AFM and TEM data.

For the first time, we demonstrate the effectiveness of the light activated antimicrobial surface against a range of micro-organisms including: yeast, viruses, filamentous fungi and fungus-like organisms. Previous studies having concentrated solely on bacteria. The efficacy of the material against these organisms provides promise for future applications in reducing the transmission of nosocomial infections in the hospital environment and reducing the numbers of pathogens on high-touch surfaces in public spaces.

## Methods

### Microorganisms and culture methods

*Staphylococcus epidermidis* RP62A was cultured on Mannitol Salt Agar (MSA) (Oxoid Ltd., UK) and incubated at 37 °C for 48 hours.

*Saccharomyces cerevisiae* was cultured on Yeast Extract Peptone Dextrose (YPD) (Sigma-Aldrich, USA) agar and incubated at 25 °C for 48 hours.

MS2 Bacteriophage (ATCC® 15597-B1) was propagated by adding 1 ml of Trypticase Soy Broth (TSB) (Becton, Dickinson & Co., USA) to the freeze dried vial and vortexing to mix. *E*. *coli* C-3000 (ATCC® 15597) in TSB was spread over on Tryptone Soya Agar (TSA) (Oxoid Ltd., UK) plates and allowed to air dry for 1 hour. Serial dilutions of the phage suspension in TSB were spread on the plates and incubated for 16 hours at 37 °C.

*Pythium ultimum* and *Botrytis cinerea* were cultured on Potato Dextrose Agar (PDA) (Oxoid, Ltd., UK) and grown for five days at 23 °C.

### Preparation of antimicrobial surfaces

The silicone samples (1 cm^2^) were synthesised as described in the literature^5^. In brief, the test samples were developed by immersing medical grade silicone MED82-5010-40 (Polymer Systems Technology Ltd, UK) in a 9:1 acetone (VWR, UK): 2 nm gold nanoparticles (BBI International Ltd. UK) solution, saturated with methylene blue hydrate (700 mg/L, Sigma-Aldrich, UK) for 72 h. After air-drying (24 h) and washing the samples with distilled water, they were immersed in an aqueous crystal violet solution (0.001 mol dm^−3^, 72 h) and subsequently washed. Control samples were prepared by immersing silicone sections in a 9:1 acetone: water swelling solution for 72 h, after which the samples were air-dried (24 h) and washed.

### Assay of the antimicrobial activity of surfaces

The microbial suspensions were tested on the surfaces inside a sterile humidity chamber, consisting of a Petri dish lined with moistened filter paper, to protect the suspension from contamination and prevent it from drying out under illumination.

Triplicates of the 1 cm^2^ test and control surfaces were gently sterilised with 70% ethanol wipes and placed onto the glass slides of the humidity chambers. The surface was inoculated with 20 µl of the microbial suspension and covered with a 2.2 cm^2^ glass cover slip. 15.

***S***. ***epidermidis*** **RP62A** was cultured in BHI broth for 16 hours at 37 °C/200 rpm. The test was carried out on cells in stationary phase. A bacterial suspension in phosphate buffered saline (PBS) was standardised to an optical density (600 nm) of 1.7 and diluted to 10^−3^ resulting in a suspension of approximately 10^6^ cells per ml. The suspension was applied to the surfaces as described above. Exposure times of 1 hour, 1.5 hours and 3 hours were tested. The cells were resuspended in PBS by vortexing for one minute and viable cells enumerated by performing serial 10-fold dilutions and subsequent culture on MSA for 48 h at 37 °C.

***S***. ***cerevisiae*** was cultured in YPD broth for 24 hours at 25 °C/150 rpm. The cells used in the bioassay were in stationary phase. A suspension in PBS was standardised to an optical density (600 nm) of 2.25 and diluted 10^−3^ resulting in a suspension of approximately 10^5^ cells per ml. The suspension was applied to the surfaces as described above. Exposure times of 3 hours and 7.5 hours were tested. The cells were resuspended in PBS by vortexing for one minute and viable cells enumerated by performing serial 10-fold dilutions and subsequent culture on YPD for 48 h at 25 °C.

The titre of the **MS2 Bacteriophage** stock was determined by counting the number of plaques formed in the bacterial lawn at the appropriate dilution factor. The suspension was applied to the surfaces as described above. Exposure times of 4 hours and 8 hours were tested. The phage was then ten-fold serially diluted in phage buffer twice, mixed with *E*. *coli* culture in 0.5% agar, which was then gently mixed and then immediately poured over pre-warmed TSA plates. Once the top agar was set, the plates were incubated at 35 °C for 16 hours. The titre of each sample was determined by counting the number of plaques formed in the bacterial lawn at the appropriate dilution. Plates that showed plaques in the bacterial lawn where then scraped off into phage buffer (1 ml 1 M Tris, pH 7.5, 1 ml 1 M MgSO_4_, 0.4 g NaCl, 98 ml ddH_2_O, 100 μl 1 mM CaCl_2_) and filter sterilized using a 0.22 μm filter head. The phage stocks were maintained at −80 °C in phage buffer with 15% (v/v) glycerol.

The activity of the surfaces on **filamentous fungus**, ***B***. ***cinerea***, and the fungus like organism, ***P***. ***ultimum*** was tested by placing a 7 mm cork borer inoculum in the centre of a PDA plate. Four test surface squares were placed evenly, exactly 1.5 cm from the inoculum to ensure that each square received equal exposure to the fungi. The PDA plates were incubated at 23 °C under an 8 W fluorescent tube light with an intensity of 2600-3500LUX for 5 days. Replicates with control squares in light, and test and control squares at in darkness were also incubated at 23 °C for 5 days. Subsequently, each square was photographed under a dissecting microscope (Leica EZ4; Leica Microsystems GmbH, Germany) using an 8MP camera (Moto G, Motorola Inc., USA). Each image was cropped to 10 cm^2^ using PAINT.NET software package (dotPDN LLC., USA) and the contrast and brightness adjusted to best show the presence of hyphal growth on the surface. A 10 × 10 grid was overlaid onto the image and grid squares that were judged to have over 50% hyphal coverage were marked as positive. Antimicrobial activity of the test surface in light was assessed by comparing the average hyphal growth over the top of the surface with the experimental controls.

### Statistical analysis of the antimicrobial effect

The *S*. *epidermidis*, *S*. *cerevisiae* and MS2 Bacteriophage data were analysed for statistical significance using the Mann-Whitney U Test. The filamentous organism data were tested for statistical significance using the ANOVA test. The IBM SPSS Statistics 20 software package (IBM Corp., USA) was used to perform both tests.

### Imaging of the control and test surfaces

The surfaces were observed using a Nanosurf Easyscan 2 AFM (Nanosurf Inc., USA). The AFM settings were: tapping mode, tip: NCLR, I-gain 10000, P-gain 5000. Thirty random 10 µm^2^ sections of each silicone surface were scanned and the mean surface roughness of each silicone surface was calculated using the Nanosurf Easyscan 2 software package (Nanosurf Inc., USA). The mean surface roughness of the test and control silicone surfaces were analysed for statistical significance using independent samples t-test on the IBM SPSS Statistics 20 software package (IBM Corp., USA). For all analyses, P ≤ 0.05 was considered statistically significant.

### Measuring leaching of photosensitizers from antimicrobial surface

A test square that had been sterilised with a 70% ethanol wipe was suspended in 3 ml PBS in a sterile 7 ml polystyrene bijou tube and vortexed for 5 seconds. The bijou tube was illuminated for 90 minutes under a 28 W compact fluorescent lamp, held 30 cm above the samples using clamp stands, and at an intensity range of 3600-4000 LUX. A replicate was also kept in darkness under a box for 90 minutes. The absorbance peaks at 590 nm and 651 nm of both PBS samples was measured using a UV-1800 Spectrometer (Shimadzu Corporation). The experiment was performed in triplicate and the data were analysed for statistical significance using the independent samples t-test on IBM SPSS Statistics 20 software package (IBM Corp., USA).

### Imaging of *S*. *epidermidis* RP62A after treatment with antimicrobial surface

#### Optical Microscopy

*S*. *epidermidis* RP62A colonies on MSA were suspended in 3 ml ddH_2_O to McFarlane 1.0. A test square was suspended in 1 ml of the bacterial suspension and left in the dark for 10 min. After 10 minutes, 10 µl aliquots of the treated and untreated bacterial suspensions were spotted onto glass slides with cover slips applied and observed under an optical microscope with a digital camera. A replicate using sterile ddH_2_O were also performed. Aliquots (10 µl) of the untreated bacterial suspension were also spotted onto test and control surfaces with a cover slip applied over them, and observed under an optical microscope. Replicates using sterile ddH_2_O in place of the bacterial suspension were also performed.

#### Atomic Force Microscopy

Samples (150 µl) of *S*. *epidermidis* RP62A that had been treated as described previously were mixed with 1:1 3% glutaraldehyde and left at 25 °C for two hours. After incubation, 50 µl of the solution was spread over a glass slide and allowed to air dry. Once dry, the slide was submerged in 90% ethanol for 5 minutes. The glass slides were imaged using Bruker ICON AFM (Bruker Inc., USA). The settings for AFM were: Scanayst in air mode, tip: RTESP. Cross section analysis of the images was performed using Nanoscope V 1.5 software package (Bruker Inc., USA).

#### Transmission Electron Microscopy

A drop of the 1:1 glutaraldehyde-*S*. *epidermidis* RP62A samples (section 10.2) was spotted onto a formvar/carbon on 200 mesh gold TEM grid (Agar Scientific Ltd., UK) and left for 45 minutes at room temperature. A Petri dish lid was placed over the grid during this time to protect it from dust. After 45 minutes, the excess inoculum was removed from the grid by picking it up gently with tweezers and touching it against filter paper. The grid was then held at 45° with tweezers and a drop of 1% methylamine tungstate dye in H_2_O was dropped across the surface of the grid. After 1 second, the excess dye was removed with filter paper. Images were taken using a Philips CM12 Transmission Electron Microscope operating at 80 kV at The Eastman Dental Institute, UCL.
